# Identifying Social Withdrawal (*Hikikomori*) Factors in Adolescents: Understanding the *Hikikomori* Spectrum

**DOI:** 10.1007/s10578-020-01064-8

**Published:** 2020-09-21

**Authors:** Yukiko Hamasaki, Nancy Pionnié-Dax, Géraldine Dorard, Nicolas Tajan, Takatoshi Hikida

**Affiliations:** 1 Faculty of Contemporary Society, Kyoto Women’s University, 35, Kitahiyoshi-cho, Imakumano, Higashiyama-ku, Kyoto, 605-8501 Japan; 2 Shigasato Hospital, Shiga, Japan; 3 Child and Adolescent Psychiatry Department, EPS ERASME, Antony, France; 4 Université de Paris, LPPS, 92100 Boulogne-Billancourt, France; 5 Psychopathology and Psychoanalysis Laboratory, Graduate School of Human and Environmental Studies, Kyoto University, Kyoto, Japan; 6 Laboratory for Advanced Brain Functions, Institute for Protein Research, Osaka University, Osaka, Japan

**Keywords:** *Hikikomori*, Social withdrawal, Adolescence, Early intervention, Mental health

## Abstract

*Hikikomori* is a Japanese term for social withdrawal, ranging from complete inability to venture outdoors to preferring to stay inside. The prevalence of *hikikomori* is high, up to 1.2% of the Japanese population, but only few studies have examined its emergence in adolescents. Therefore, we sought to identify environmental and psycho-behavioral characteristics related to *hikikomori* during adolescence. Parents of middle school students who underwent psychiatric outpatient treatment for *hikikomori* (*n* = 20) and control group parents (*n* = 88) completed the Child Behavior Checklist to evaluate their child’s psycho-behavioral characteristics and novel scales to evaluate environmental characteristics and *hikikomori* severity. Scores for all eight Child Behavior Checklist subscales were significantly higher in the experimental group. Multiple regression analysis revealed that “anxious/depressed,” “somatic complaints,” “lack of communication between parents” and “overuse of the Internet” were significant predictors of *hikikomori* severity. These findings can help identify individuals who are at risk of developing *hikikomori*.

**Electronic supplementary material:**

The online version of this article (10.1007/s10578-020-01064-8) contains supplementary material, which is available to authorized users.

## Introduction

Since the 1990s, social withdrawal (hereinafter referred to as *hikikomori*) emerged as a serious psychosocial problem in Japan [1–5]. Beginning in 2000, the number of studies on *hikikomori* grew, mainly in the field of sociology [6–8]; however, in psychiatric journals, the concept was first mentioned in 2010 [1, 2, 9, 10]. The term *hikikomori* is often translated as “social withdrawal” internationally, but in Japanese, the term refers to both the phenomenon and to the socially withdrawn person.

In recent systematic reviews, *hikikomori* has been defined as a 6-month or longer period of living at home and avoiding social situations and relationships, along with significant distress and impairment [1, 9]. According to epidemiological surveys, the lifetime prevalence of *hikikomori* among young adults is approximately 1.2% in Japan [10]. Onset typically occurs during adolescence or early adulthood and, on an average, it takes 4 years before symptoms are addressed clinically; the treatment often involves circadian rhythm correction, cognitive behavioral therapy, and symptomatic drug therapy [3, 11].

Almost half of the patients with *hikikomori* who visit health centers are diagnosed with mood and anxiety disorders, personality disorders, sleep loss disorders, pervasive developmental disorders, or schizophrenia [10–14]. The question of whether we can distinguish *hikikomori* from other psychiatric disorders, particularly social anxiety disorders, is pertinent and some research has attempted to delineate the differences of interest. Reports from the Japanese Cabinet Office; Ministry of Health, Labor and Welfare; and numerous articles since 2010 show that social anxiety disorders and agoraphobia only apply to a subcategory of *hikikomori* cases [4, 15–19]. About 19% of social anxiety disorder patients can also be classified as *hikikomori* [20] and about 18% of hikikomori patients are also diagnosable with social anxiety disorder [21]. Hence, it is epidemiologically clear that there is duplication but the two conditions are not identical. However, specific features unique to *hikikomori* are yet to be elucidated; therefore, *hikikomori* is not yet included in the DSM-5.

*Hikikomori* was thought of as a concept that refers to both distress and to a cultural syndrome unique to Japan [3, 9, 19, 22], however, recent international surveys have shown that *hikikomori* is also found among different populations of the world, including South Korea, India, Australia, Bangladesh, Iran, Taiwan, Thailand, and the United States [2, 9, 19]. Additional cases have been subsequently reported in Oman [23], France [12, 22, 24, 25], Brazil [26], Hong Kong [27], Spain [13, 28, 29], China [30], and Canada [31, 32]. The phenomenon of *hikikomori* is considered to be a boundless and global syndrome found across many cultures [3, 33], but notably, is more common in urban areas [19] and high-income, developed countries [2].

Compared to studies of *hikikomori* in adults, fewer studies have been conducted with adolescents, although a strong relationship between *hikikomori* and refusal to attend school has been established [34–36]. Adolescence is a developmental period that has a significant influence on later socio-academic achievement and often marks the onset of psychiatric symptoms [37]. Understanding what triggers *hikikomori* is critical for secondary prevention, early intervention, and for minimizing the risk of chronicity [10]. Considering that *hikikomori* tends to persist once it develops [15, 16], it greatly affects the national health, welfare, and workforce [14]. Therefore, it is imperative to elucidate the etiology of *hikikomori* to establish prevention and treatment methods for this worldwide phenomenon.

Given that epidemiological studies on *hikikomori* are still scarce, many of the related factors remain unknown. The Cabinet Office of Japan conducted several well-designed studies on young people’s attitudes (Fact-finding Survey on Social Withdrawal, SYPA) that contained valuable information about socio-demographic and mental health factors within this population; although, the data were not fully analyzed for correlations [15, 16, 38, 39]. Hence, factors associated with the etiology of *hikikomori* were not investigated, and no intervention methods were discussed. The SYPA data also included a wide age range (15–39 years) making it difficult to gain a clearer understanding of the characteristics associated with *hikikomori* during adolescence.

In the SYPA surveys, refusal to attend school was mentioned as the most frequent trigger of *hikikomori* [16, 38, 39]. Similarly, a recent secondary analysis study using the SYPA data reported that the history of dropping out of school was an important factor associated with *hikikomori* [14]. Notably, school refusal, along with mental health problems, increases significantly in middle school students [40, 41]. A recent systematic review identified maladaptive parenting and family dysfunction as critical factors in the development of *hikikomori,* specifically among adolescents [42]. Therefore, middle-school age should be considered as a “critical period” (also from a neurodevelopmental perspective) [37], which is vital for early detection and intervention.

Therefore, in the present study, we focused on observing middle school students and investigated the relationship between individual psycho-behavioral characteristics and the degree of severity of *hikikomori*. We also assessed the environmental situations with the purpose of identifying the factors related to the occurrence and severity of *hikikomori* during adolescence.

## Methods

### Participants

Our study targeted psychiatric outpatients, aged 12–15 years (seventh to ninth graders), who visited an adolescent outpatient clinic between December 2014 and November 2015. These participants were being primarily treated for *hikikomori* (n = 20; 10 of each sex; mean age ± standard deviation (SD) = 14.1 ± 1.1). We also recruited a healthy control group (n = 88; 56 boys; mean age ± SD = 14.0 ± 0.9). Among the clinical patients visiting the hospital chiefly for *hikikomori*, we targeted those who met the Cabinet Office’s definition; at least 6 months of a person exhibiting either “quasi-*hikikomori*” (*i.e.*, going out only to engage in hobbies) or *hikikomori* in the narrow sense (*i.e.*, from almost never going out of one’s room to going out to nearby convenience stores) [16]. Additionally, this definition excludes those diagnosed with schizophrenia and/or physical illness. We only included participants who, according to the DSM-IV-TR, did not meet the criteria for either Axis I or Axis II mental disorders.

For the healthy control group, we primarily recruited the siblings of student volunteers at Kyoto Women’s University, using a snowball sampling method (where respondents recommend additional eligible participants) and matched them with members of the patient group according to gender and age. No significant statistical differences were observed between the groups based on these characteristics (Supplemental Table 1). The exclusion criteria for healthy volunteers were the same as above, namely no diagnosis of schizophrenia, physical illness or Axis I or II mental disorders. All participants in the patient and healthy control groups received information regarding the survey and all parents and children provided consent to participate. Parents signed written consent forms for the participation of minors. Our study was carried out following a review, and permission was granted by the Clinical Study Ethical Review Board of Kyoto Women’s University.

### Assessing the Severity of *Hikikomori*

Patients for the *hikikomori* group were pre-selected (on the basis of the treatment for the condition at an outpatient clinic). Since no DSM criteria currently exist and no formal diagnosis could be issued, the severity of each case needed to be established. We created a novel scale for evaluation (included in the “Appendix 1”), which was administered to the parents of both groups.

Based on the target age (school age) and the definition of *hikikomori* proposed in the report released by the Cabinet Office [15, 16, 38, 39], we designed our evaluation scale to comprise two items: (a) absenteeism from school and (b) going out; the latter is defined as: “the child went out either alone or with friends (but unaccompanied by family members) to shop, engage in sports, and/or socialized with friends.” The Cabinet Office’s report also identifies people who relate with individuals with *hikikomori* and also prefer to stay inside their homes and defines them as the “*hikikomori* affinity group” (Definition of *hikikomori*. 2016: 9–11) [16]. The survey did not recognize *hikikomori* as an independent clinical category, but rather as a continuous spectrum that included both the healthy and the affinity groups. This view set the tone for subsequent research on *hikikomori.* Therefore, in the present study, we attempted to follow the spectrum concept and evaluated our participants, who ranged from healthy to severe, using the same *hikikomori* scale.

Evaluations were conducted by asking parents to consider the most frequent occurrence of (a) and (b) during the past 6 months. Responses were provided on a 5-point scale, ranging from 0 (“Not at all”) to 4 (“Always”). For item (b), the numerical values were reverse scored and then combined with the scores for item (a). The total score represented the degree of severity of *hikikomori*, with a higher score indicating more severity*.* We calculated Cronbach’s α as 0.703 upon conducting a reliability analysis.

### Measuring Environmental Factors

To investigate which environmental factors could be related to the occurrence and severity of *hikikomori* during adolescence, we created another novel evaluation scale to measure the following: (1) parental mental health, (2) parental physical conditions, (3) communication between parents and child, (4) communication between parents, (5) conflict between parent and child, (6) conflict between parents, (7) financial status, (8) communication with the community, (9) overuse of the Internet. Evaluations were conducted by asking parents of both groups to consider the circumstances over the past 6 months. Responses were provided on a 5-point scale (included in the “Appendix 1”).

### Measuring Psycho-Behavioral Characteristics

The parents of participants in the *hikikomori* and control groups answered all questions in the Child Behavior Checklist (CBCL4-18) [43, 44] to evaluate their child’s psycho-behavioral characteristics. The CBCL was developed by Achenbach and colleagues to comprehensively evaluate children’s emotional and behavioral problems [45]. Based on the raw scores from 118 problem-behavior questions featured in the CBCL4-18, the scores of 11 scales were calculated: eight syndrome subscales (*i.e.*, withdrawn, somatic complaints, anxious/depressed, social problems, thought problems, attention problems, delinquent behavior, and aggressive behavior), an internalizing scale, an externalizing scale, and a total score scale. The scores of these 11 scales were converted into standardized *t*-scores based on country-specific standard values [46–50]. The CBCL has become a major research tool widely used in retrospective, cohort, and meta-analysis studies [51–55]. In the present study, we chose to use the CBCL to identify in detail the subclinical characteristics and symptoms related to *hikikomori*.

### Statistical Analysis

We produced descriptive statistics and *t*-tests to determine between-group differences on the *hikikomori* severity scale, nine environmental scales, eight CBCL syndrome subscale *t*-scores, and the total CBCL *t*-score. Effect size (Cohen’s *d*) was calculated to ensure that the sample size was sufficient. We considered *d* > 0.5 as medium effect size and *d* > 0.8 as large effect size [56]. The dependent variables were approximately normally distributed within each group.

To identify factors related to *hikikomori* severity, we conducted multiple regression analysis with the severity of *hikikomori* as the dependent variable and demographic variables (gender, age), CBCL subscale *t*-scores, and the nine environmental factors as the predictor variables (n = 108). Two models were calculated: the first model (Model 1) was adjusted for all explanatory variables, and multicollinearity verification was performed using variance inflation factor (VIF) statistics. We then conducted a second multiple regression analysis (Model 2) excluding variables exhibiting VIF > 2.0 and variables with a low contribution (β < 0.01) to the first model. All analyses were performed using SPSS (v22) software for Windows and the significance level was set at *p* < 0.05.

## Results

### Descriptive Statistics and Comparisons of *Hikikomori* Severity*,* Environmental Factors, and CBCL Scores

The results of descriptive statistics are presented in Tables 1 and 2. *Hikikomori* severity was, as expected, significantly higher in the *hikikomori* patient group (*p* < 0.001, *d* > 0.8). With regard to environmental factors, in the *hikikomori* patient group, “parental psychiatric disorders” (*p* < 0.05, *d* > 0.8), “conflict between parent and child” (*p* < 0.001, *d* > 0.8), and “overuse of the Internet” (*p* < 0.05, *d* > 0.5) were all significantly higher than the control group, while “communication between parents” (*p* < 0.01, *d* > 0.8) was significantly lower. In the *hikikomori* patient group, mean values for the total CBCL score and syndrome subscales were significantly higher than those of the control group. In the *hikikomori* patient group, the total CBCL score was in the clinical range, while all syndrome subscale scores were in the subclinical range.

**Table 1:** Comparisons of severity of *hikikomori* and environmental factors scores (original scales) between *hikikomori* and control groups

	*Hikikomori* Group	Control Group			
	Mean ± SD (SE)	Mean ± SD (SE)	*t*-value	*p*	*d*
Severity of *hikikomori*	4.47 ± 1.48 (0.33)	0.98 ± 1.18 (0.12)	11.29	0.000***	2.82
Environmental factors					
Parent’s psychiatric disorder	0.70 ± 1.26 (0.28)	0.09 ± 0.51 (0.05)	2.12	0.046*	0.86
Parent’s physical disorder	0.35 ± 0.87 (0.19)	0.63 ± 1.06 (0.11)	− 1.12	0.265	0.27
Communication between parents and child	3.30 ± 0.73 (0.16)	3.56 ± 0.69 (0.07)	− 1.54	0.124	0.37
Communication between parents	2.20 ± 1.36 (0.30)	3.30 ± 0.92 (0.09)	− 3.45	0.002**	1.09
Conflict between parents and child	1.70 ± 1.12 (0.25)	0.79 ± 0.85 (0.09)	3.99	0.000***	1.01
Conflict between parents	1.15 ± 1.26 (0.28)	0.64 ± 0.88 (0.09)	2.10	0.106	0.53
Economic status	2.92 ± 1.21 (0.27)	2.92 ± 1.01 (0.12)	0.01	0.986	0.00
Communication with the community	2.70 ± 1.21 (0.27)	2.70 ± 1.14 (0.12)	− 0.01	0.987	0.00
Overuse of the internet	3.20 ± 0.95 (0.21)	2.48 ± 1.18 (0.12)	2.50	0.014*	0.63

*T-*test comparisons

^*^p < 0.05, **p < 0.01, ***p < 0.001; *d* = Effect size (Cohen’s *d*)

**Table 2:** Descriptive statistics and comparisons of CBCL *t*-scores

	*Hikikomori* group	Control group			
	Mean ± SD (SE)	Mean ± SD (SE)	*t*-value	*p*	*d*
Summary scale^‡^					
Total score	65.82 ± 6.06 (1.34)	46.92 ± 13.01 (1.38)	9.77	0.000***	1.57
Eight syndrome subscales^§^					
Withdrawn	68.48 ± 9.48 (2.12)	52.82 ± 5.35 (0.57)	7.13	0.000***	2.49
Somatic complaints	63.19 ± 8.23 (1.84)	51.81 ± 6.78 (0.72)	5.75	0.000***	1.61
Anxious/depressed	65.56 ± 7.58 (1.69)	51.76 ± 6.90 (0.73)	7.92	0.000***	1.96
Social problems	59.85 ± 6.68 (1.49)	52.76 ± 4.36 (0.46)	4.52	0.000***	1.46
Thought problems	58.15 ± 10.23 (2.28)	50.70 ± 2.64 (0.28)	3.23	0.004**	1.51
Attention problems	60.22 ± 5.83 (1.30)	53.13 ± 5.37 (0.57)	5.24	0.000***	1.30
Delinquent behavior	58.68 ± 6.67 (1.49)	52.49 ± 5.06 (0.53)	3.90	0.001**	1.15
Aggressive behavior	58.50 ± 4.98 (1.11)	53.17 ± 5.48 (0.58)	3.99	0.000***	0.99

*T*-test comparisons

*p < 0.05, **p < 0.01, ***p < 0.001; ^‡^ = 63 < clinical range of total score; § = 70 < clinical range of syndrome subscales; *d* = Effect size (Cohen’s d)

### Associations Between *Hikikomori* Severity and Demographic Variables, Environmental Factors, and CBCL Subscale Scores

To identify factors related to *hikikomori* severity, a multiple regression analysis was conducted using “severity of *hikikomori”* as the dependent variable and demographic variables (gender and age), the eight CBCL syndrome subscale scores, and the nine environmental factors as the independent variables (Model 1, Supplemental Table 2). The variable that most contributed to the severity of *hikikomori* was the CBCL syndrome subscale “withdrawn.” However, after estimating VIF, “withdrawn” was removed as an independent variable as it exhibited multicollinearity.

Therefore, we attempted to use variables with VIF < 2.0 and identified “independent” factors related to *hikikomori* severity with multiple regression analysis. The following items were excluded from our explanatory variables: withdrawn, social problems, thought problems, attention problems, delinquent behaviors, aggressive behaviors, and conflict between parent and child. Furthermore, variables with a low contribution (β < 0.01) were also omitted, resulting in conflict between parents also being excluded from the explanatory variables. See Table 3 for Model 2 results. Among the selected independent variables, “somatic complaints,” “anxious/depressed,” “overuse of the Internet,” and “lack of communication between parents” were significantly associated with *hikikomori* severity.

**Table 3:** Multiple linear regression analyses with demographic variables, CBCL subscales, and environmental factors to predict *hikikomori* severity (Model 2)

Independent variables	Beta	*p*	VIF
Sex (Female)	0.128	0.089	1.076
Age	− 0.076	0.320	1.139
Somatic complaints	0.277	0.001**	1.383
Anxious/depressed	0.311	0.000***	1.455
Parents’ psychiatric disorder	0.119	0.167	1.426
Parents’ physical disorder	− 0.105	0.171	1.138
Communication between parents and child	− 0.058	0.474	1.270
Communication between parents	− 0.190	0.034*	1.537
Economic status	0.081	0.361	1.525
Communication with the community	− 0.031	0.716	1.400
Overuse of the internet	0.216	0.006**	1.148

*p < 0.05, **p < 0.01, ***p < 0.001

^†^Multiple regression model statistics: R2 = 0.509. ANOVA *p* < 0.001

## Discussion

There are limited studies pertaining to the etiology of *hikikomori*. Our study aimed to identify factors associated with the occurrence and severity of *hikikomori* during early adolescence, which is a critical period in the development of the disorder.

First, we developed a novel scale that could measure the severity of *hikikomori* and accurately identify those suffering from it, by comparing the results with those of control participants. This scale was based upon the findings of other research that identified school absenteeism and being house bound as two critical symptoms. We believe this scale can be useful but will require further validation by other studies, especially to improve upon its specificity as there may be some crossovers with mood disorders and agoraphobia.

### Factors Associated with the Occurrence of *Hikikomori*

Previous research has found that individuals who exhibit *hikikomori* are more likely to be male [12, 14], however, gender was not significantly related to *hikikomori* severity in our study.

Our investigation of environmental factors that may be associated with the occurrence of *hikikomori* found that the prevalence of psychiatric disorders among parents was significantly higher in the *hikikomori* group. This indicated that there may be some genetic predisposition; perhaps related to stress tolerance, coping ability, or resilience; preventing adolescents with *hikikomori* from adequately coping with stressors such as interpersonal problems at school or poor academic performance. A recent preliminary study has shown blood biomarkers uric acid and high-density lipoprotein cholesterol as possibly correlated with an underlying biological pathology of *hikikomori* [57]. Individual psychological factors including interpersonal problems [14], coping difficulties, conflicting demands, reduced autonomy [58], low self-esteem [34], and a predisposed introverted personality [31] have been shown to play some role in *hikikomori* propensity. However, the extent to which these underlying vulnerabilities depend on a biological foundation requires further research. The novel scale we designed to measure the environmental factors also requires further testing and validation.

We also found that the *hikikomori* group had significantly lower scores for communication between parents and significantly higher scores for conflict between parent and child. Overuse of the Internet was also significantly higher in the clinical group. These could be important risk factors for *hikikomori* but could also be a result of the *hikikomori* itself. When personal stress and a negative family environment are added to a nonspecific vulnerability, signs of *hikikomori* could emerge along with adaptation issues. Similarly, maladaptation (in the form of *hikikomori*) may increase conflicts between parent and child and perhaps eventually lead to decreased communication between parents should they become overwhelmed. Familial factors, including an absent father, overdependence between mother and child [3], highly educated parents, and maternal panic disorder [59] have all been associated with *hikikomori*.

Overuse of the Internet may merely be a product of the limited available things to do when confined to the home, and more investigation is needed to uncover the relationship between Internet use and *hikikomori*, specifically to ascertain whether Internet use actively worsens symptoms or whether it is purely a recreational activity replacing social interaction.

Our CBCL results showed that middle school *hikikomori* patients had significantly higher mean scores for all the syndrome subscales and the total score, as compared to the control group. Although the total mean CBCL score for the *hikikomori* group was in the clinical range, all eight syndrome subscales were subclinical. This may be interpreted as follows: each of these psychiatric signs associated with *hikikomori* may not be considered clinically serious when considered alone; however, the combination may warrant psychiatric consultation. Given that there is no distinctive psychiatric sign that is specific to “clinical” *hikikomori*, as compared to other psychiatric conditions, there may be no single strong predictor that could be used for early detection. Rather, its occurrence will need to be judged by analyzing a combination of features that will change along a spectrum that has “severe *hikikomori*” at its one extreme [15, 16, 38, 39]. Based on our findings, it is unlikely that a specific vulnerability is the foundation of this condition and it is unclear whether the comorbidities reported thus far [10–13] may be secondary to the development of *hikikomori.*

### Factors Associated with the Severity of *Hikikomori*

We used multiple regression analyses to investigate environmental factors and psychological characteristics that may be associated with *hikikomori* severity. The CBCL syndrome subscale “withdrawn” was found to contribute the most to *hikikomori*. This subscale evaluates the psychological tendencies of *hikikomori* and is one way to quantify “affinity for *hikikomori*,” as mentioned in the Cabinet Office reports [15, 16]. However, since we tried to investigate psychological factors that may have played a role in social withdrawal (*hikikomori* affinity) the “withdrawn” phenotype was too centrally involved to be useful and thus could not function as an independent variable in our model due to multicollinearity issues.

The results from our cross-sectional multiple regression analysis revealed that the following independent variables were correlated with *hikikomori* severity: “somatic complaints,” “anxious/depressed,” “overuse of the Internet,” and “lack of communication between parents”. It is interesting to note that “lack of communication between parents” was a correlate but “conflict between parents” was not. Could this indicate that regardless of whether parents frequently quarreled, more communication between parents could be a protective factor for adolescents with a tendency toward *hikikomori*? A more sensitive measure of the quality of the communication, such as the Family Assessment Device [60], would be useful to interrogate this further.

It is also not easy to tell whether anxiety and depression are triggers for *hikikomori* or simply co-occur. They have been identified as factors in other studies, but the exact relationship remains unclear [11, 12].

The relationship between somatic complaints and *hikikomori* is also unclear. Somatization could be related to nonspecific genetic vulnerabilities mentioned above (*e.g.* low stress tolerance). As a result of somatization, those with early *hikikomori* may frequently visit pediatricians about undefined complaints, which presents an opportunity for early detection. Although early screening for *hikikomori* may be difficult, the symptom of “school refusal” seems to be highly indicative [34–36]. One must also consider others on the *hikikomori* spectrum, who may have no problems attending school but communicate very little with people other than the members of their own families (the “*hikikomori* affinity group”).

### Therapeutic Interventions

Parents should be encouraged to control Internet use in *hikikomori* children. These recommendations should be emphasized in support programs for *hikikomori* that target middle school students. One example is an administrative intervention program in French schools that has reduced the number of adolescent drop outs, by making the school staff focus intensely on any student who is absent for 10 half-days in a month. If absenteeism persists, the case is referred to a public prosecutor, unless the situation is handled medically or socially [61]. Unfortunately, *hikikomori* sufferers are often concealed by families, stopping judiciary and administrator bodies from intervening, thereby greatly impeding prevention and intervention programs. Such situations could even be viewed as “social neglect.” Social welfare services that encourage parents to address difficulties together with their child, especially through home-visit programs, may be effective for decreasing *hikikomori* severity and duration [21, 36, 62, 63]. Pre-school developmental-behavioral screening and consecutive support programs may also help prevent early *hikikomori* [64] but adolescence is a critical period for intervention.

## Limitations and Future Work

This research is novel in that only middle school *hikikomori* patients, without any psychiatric disorders, were included in the study. Most previous studies did not distinguish between *hikikomori* co-occurring with or without other psychiatric disorders. One limitation of the present study was the small number of clinical *hikikomori* cases (n = 20), likely due to exclusion of all patients with additional psychiatric diagnoses. In this regard, larger sample sizes are needed to ensure the scientific validity of our results. In addition, CBCL assessment may have been affected by parental factors, such as psychopathological difficulties, which were more common in the *hikikomori* group. Furthermore, our participants’ ages (13–15 years) were not fully representative of the adolescence period (10–19 years), so differences may be cited in patients who are younger or older than those in our study. A study including a more heterogeneous sample in terms of age may bring some new insights. Moreover, the environmental factors were evaluated through a novel measurement scale that has not been psychometrically validated.

Despite these limitations, our results revealed some interesting avenues for further research, particularly exploring the role of communication between parents. In future studies, it would be interesting to include a standardized evaluation of family functioning to explore this association more precisely and to identify specific therapeutic goals.

In addition, sociocultural influences cannot be overlooked from our analysis, as only Japanese *hikikomori* cases were examined. However, *hikikomori* is increasingly being acknowledged as a global phenomenon and, as such, comparative cultural studies will be needed to identify universal risk factors. *Hikikomori* cases outside of Japan have been documented consistently with dozens of articles in the last 15 years referring to cases in South Korea, China, India, Australia, Bangladesh, Iran, Taiwan, Thailand, Oman, France, Brazil, Hong Kong, Spain, China, Canada and the United States [2, 9, 12, 13, 21, 23–32]. In fact, Teo and Gaw’s proposal to include *hikikomori* as a culture-bound Japanese syndrome in the DSM-5 in 2010 was not accepted [65], and several 2019 publications describe *hikikomori* as a global health problem that is “no longer culture-bound” [66, 67].

Our findings can therefore likely be extended to international cases as many similar features of *hikikomori* have consistently been reported. For instance, circadian rhythm correction is a common method of treatment in Japan, and a study of adolescent *hikikomori* sufferers in France found that many of them suffered from sleep–wake schedule disorders (73%) [12]. Many were also diagnosed with schizophrenia (37%) or mood disorders (23%), commonly seen in Japanese *hikikomori* patients as well [11, 12].

In order to ascertain which of the characteristics we have identified could be causally linked to *hikikomori,* a longitudinal study is necessary. It would be interesting to note whether increasing parent to parent communication and limiting use of the Internet might confer protection against the development or worsening of *hikikomori*.

Ultimately, to develop effective prevention and intervention systems that are adapted to *hikikomori* severity, it is necessary to better understand the dynamic mechanisms at play, including understanding the conditions that result in *hikikomori* compared to co-morbid conditions. It is estimated that 30% of *hikikomori* cases last more than 3 years and 15% more than 7 years [16]. This has a severe impact, not only in the lives of adolescents and their families, but also on the nation’s health, labor force, welfare, and economy. The importance of research into this debilitating condition cannot be overstated.

## Summary

This study examined characteristics surrounding the phenomenon of *hikikomori* in adolescents, since only few studies have examined this age group. We sought to identify environmental and psycho-behavioral characteristics related to *hikikomori* to better understand the etiology. Middle school students who underwent psychiatric outpatient treatment for *hikikomori* were recruited for the patient group and age and sex matched controls were recruited for the control group. Parents of both groups completed the Child Behavior Checklist to evaluate their child’s psycho-behavioral characteristics. Novel scales, also completed by the parents, were used to evaluate environmental characteristics and *hikikomori* severity. Scores for all eight Child Behavior Checklist subscales were significantly higher in the patient group. Multiple regression analysis revealed that “anxious/depressed,” “somatic complaints,” “lack of communication between parents” and “overuse of the Internet” were significant predictors of *hikikomori* severity. These findings can help identify individuals who are at risk of developing *hikikomori*.

### Electronic supplementary material

Below is the link to the electronic supplementary material.

Supplementary file1 (DOCX 39 kb)

Supplementary file2 (DOCX 41 kb)

## Appendix 1

### Assessment Scales of *Hikikomori* Severity

1. During the past 6 months, your child has been absent from school:
  Responses were provided on a 5-point scale, ranging from 0 (“Never”) and 2 (“Sometimes”) to 4 (“Completely”).
2. During the last 6 months, your child went out, either alone or with friends (*i.e.*, unaccompanied by family members) to shop, engage in sports, and/or socialize with friends:

Responses were provided on a 5-point scale, ranging from 0 (“Never”) and 2 (“Sometimes”) to 4 (“Regularly”).

### Assessment Scales of Environmental Factors

Responses were provided on a 5-point scale, ranging from 0 (“Not at all”) to 4 (“Always”), except for item #7, which was evaluated on a 5-point scale ranging from 0 to 4, representing “Extremely difficult” to “Very favorable.”

1. Parental mental health: “Both or either of the parents have been treated by a psychological counselor or a psychiatrist.”
2. Parental physical conditions: “Both or either of the parents have health problems (chronic illness, surgical treatment, and/or other problems).”
3. Communication between parents and child: “There is communication between the parents and their child.”
4. Communication between parents: “There is communication between the parents.”
5. Conflict between parent and child: “There are conflicts between a parent and their child.”
6. Conflict between parents: “There are conflicts between the parents.”
7. Financial status: “The family’s financial status can be considered as…”.
8. Communication with the community: “You, as a family, are in close contact with your neighbors and the people in your community.”
9. Overuse of the Internet: “Your child spends too much time using the Internet (computers, smartphones, games consoles, and tablets, among others.)”.
