# Supplementary material for: Identifying Social Withdrawal (Hikikomori) Factors in Adolescents: Understanding the Hikikomori Spectrum
Source: Child Psychiatry Hum Dev. 2020 Sep 21;52(5):808–17. doi: 10.1007/s10578-020-01064-8 (PMC8405474; doi:10.1007/s10578-020-01064-8)
Supplement: Supplementary file 1 — Supplementary file1 (DOCX 39 kb) [file 10578_2020_1064_MOESM1_ESM.docx]

**Supplemental table 1:** Demographic variables

|  | *Hikikomori* Group | Control Group |
| --- | --- | --- |
| Sex (M/F)^a^ | 20(10/10) | 88(56/32) |
| Age (Mean±SD)^a^ | 14.1±1.1 | 14.0±0.9 |
| ^a^No significant difference between the two groups. | | |
