# Supplementary material for: Identifying Social Withdrawal (Hikikomori) Factors in Adolescents: Understanding the Hikikomori Spectrum
Source: Child Psychiatry Hum Dev. 2020 Sep 21;52(5):808–17. doi: 10.1007/s10578-020-01064-8 (PMC8405474; doi:10.1007/s10578-020-01064-8)
Supplement: Supplementary file 2 — Supplementary file2 (DOCX 41 kb) [file 10578_2020_1064_MOESM2_ESM.docx]

**Supplemental table 2:** Multiple regression analyses with demographic variables, CBCL subscales, and environmental factors predicting *hikikomori* severity

(Model 1) ^†^

| Independent Variables | Beta | *p* | VIF |
| --- | --- | --- | --- |
| Sex (Female) | .070 | .382 | 1.282 |
| Age | -.043 | .575 | 1.207 |
| Withdrawn | .346 | .005 | 2.972 |
| Somatic complaints | .211 | .020 | 1.610 |
| Anxious/Depressed | .236 | .018 | 1.953 |
| Social problems | .062 | .587 | 2.606 |
| Thought problems | -.086 | .410 | 2.198 |
| Attention problems | .077 | .522 | 2.872 |
| Delinquent behaviors | -.034 | .770 | 2.699 |
| Aggressive behavior | -.212 | .118 | 3.666 |
| Parent’s psychiatric disorder | .049 | .581 | 1.605 |
| Parent’s physical disorder | -.109 | .186 | 1.365 |
| Communication between parents and child | -.082 | .348 | 1.518 |
| Communication between parents | -.169 | .080 | 1.853 |
| Conflict between parent and child | -.022 | .852 | 2.713 |
| Conflict between parents | .002 | .982 | 1.936 |
| Economic state | .049 | .590 | 1.648 |
| Communication with the community | -.048 | .586 | 1.540 |
| Overuse of the Internet | .174 | .043 | 1.454 |

^†^ Multiple regression model statistics: R2=0.566. ANOVA p<0.001. Demographic variable (Sex, Age), CBCL syndrome scales, environmental factors were all entered in Model 1 as independent variables.
